# Pre‐Activation as a Route for Tuning the Kinetics of Mechanochemical Transformations

**DOI:** 10.1002/anie.202516632

**Published:** 2025-11-23

**Authors:** Christian Heinekamp, Tahlia M. Palmer, Dominik Al‐Sabbagh, Anastasia May, Carsten Prinz, Stefan Michalik, Adam A. L. Michalchuk, Franziska Emmerling

**Affiliations:** ^1^ Federal Institute for Materials Research and Testing (BAM) Richard‐Willstätter Str 11 Berlin 12489 Germany; ^2^ Department of Chemistry Humboldt University Berlin Berlin 12489 Germany; ^3^ School of Chemistry University of Birmingham Birmingham B15 2TT United Kingdom; ^4^ Diamond Light Source Ltd., Harwell Science and Innovation Campus Didcot Oxfordshire OX11 0DE United Kingdom

**Keywords:** Co‐crystals, In situ, Kinetics, Mechanical activation, Mechanochemistry

## Abstract

Learning to control reaction kinetics is essential for translating any chemical technology into real‐world application. Based on time‐resolved in situ powder X‐ray diffraction data, we demonstrate the opportunity to tune mechanochemical reaction rates through the pre‐activation of the starting reagents. For three model co‐crystal systems, the pre‐activation of the most stable reagent yields up to a *ca* 10‐fold increase in the reaction rate, whilst negligible kinetic enhancement is seen when the less stable reagent is pre‐activated. Moreover, we demonstrate how the polymorphic outcome of mechano‐co‐crystallization is also sensitive to pre‐activation of the starting material. Our results suggest that reproducibility of mechanochemical processes requires detailed understanding over the origin and history of reagent powders, whilst providing a new conceptual framework to design and control mechanochemical reactions.

## Graphical abstract

The kinetics of mechanochemical reactions can be markedly altered through the selective pre‐activation of starting reagents, as revealed through reaction monitoring using time‐resolved in situ powder X‐ray diffraction and ab initio density functional theory simulation.

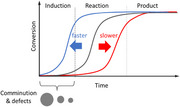

## Introduction

Mechanochemistry—inducing or sustaining physical or chemical transformations with mechanical force—is emerging as a transformative method for sustainable and green chemical processing.^[^ ^1^, ^2^ ^]^ This is exciting, as it indicates that many types of chemistry can likely be performed under solvent‐free mechanochemical conditions. Moreover, because mechanochemical processes occur under out‐of‐equilibrium conditions with local and transient high‐pressures and temperatures,^[^ ^3^, ^4^ ^]^ it is common to produce (often metastable) phases that are difficult or inaccessible by conventional synthesis routes.^[^ ^4^ ^]^

Mechanochemical transformations often defy conventional chemical intuition, exhibiting unexpected reactivity patterns and product distributions. This includes the formation of unexpected and novel products,^[^ ^5^ ^]^ unconventional kinetic profiles,^[^ ^6^, ^7^ ^]^ and even an influence of environmental or reaction parameters that are inconsequential in “conventional” chemistry.^[^ ^8^, ^9^ ^]^ Our understanding of these unique features of mechanochemical processes is still emerging,^[^ ^10^ ^]^ with most reactions still being developed through trial‐and‐error. Only a handful of examples currently attempt statistics‐led process design.^[^ ^11^ ^]^ To expedite the development of this sustainable chemical technology and facilitate the broader adoption of mechanochemistry across academic and industrial laboratories, a more detailed understanding is needed about how we can design and control these transformations.

**Scheme 1:**
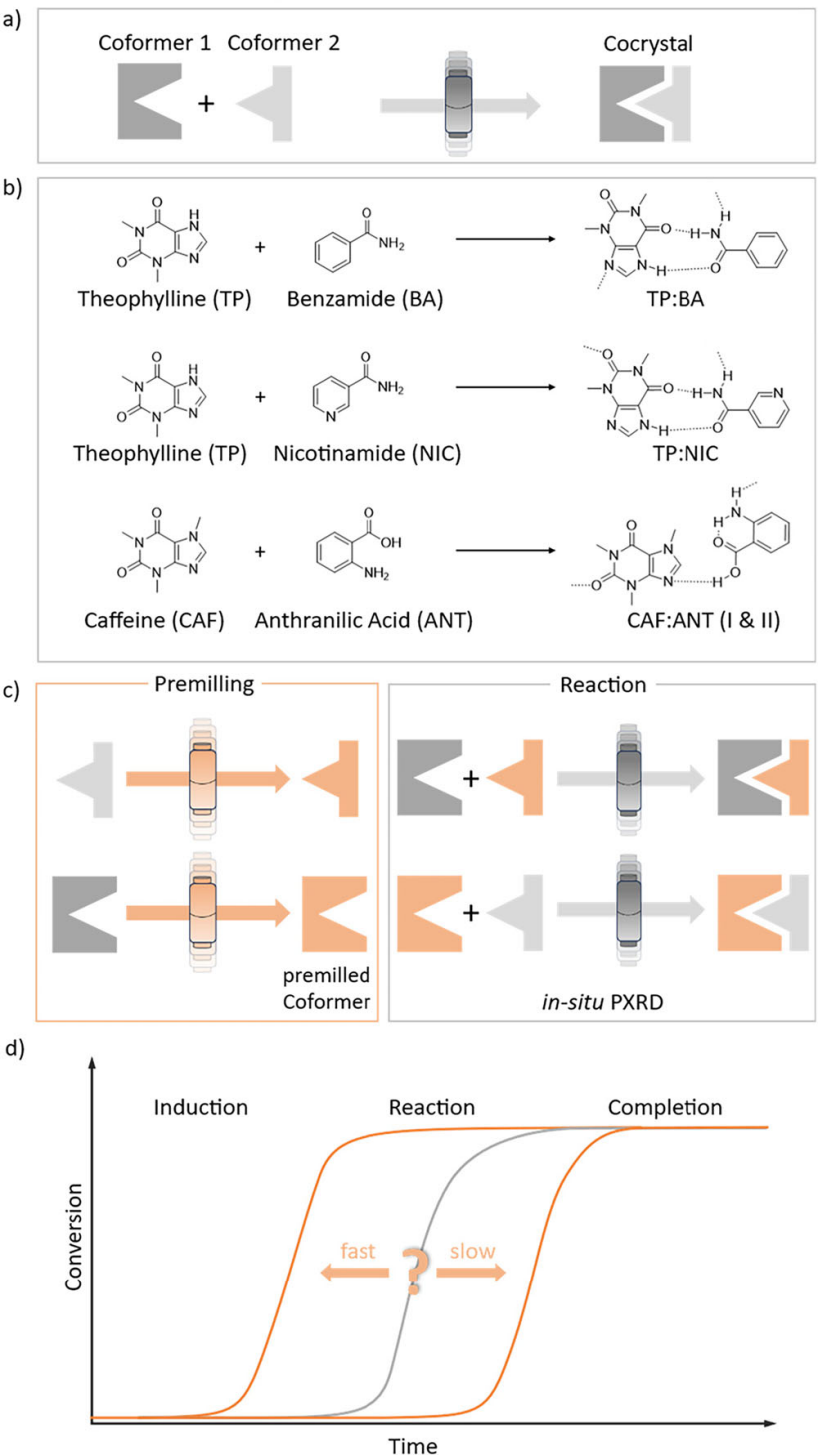
a) Co‐crystal formation via mechanochemical synthesis between two non‐pre‐activated coformers. b) Model systems: theophylline (TP) with benzamide (BA) or nicotinamide (NIC), and caffeine (CAF) with anthranilic acid (ANT). c) Pre‐activation of coformers with subsequent in situ monitoring of the reaction. d) Conversion profiles show that pre‐activation can either accelerate or delay the induction period.

Of particular interest is the unique kinetic profile commonly seen with mechanochemical transformations. Unlike chemistry in solution, mechanochemical reactions often comprise a notable induction period before the rapid onset of a transformation (reaction period), Scheme 1.^[^ ^6^, ^12^ ^]^ The length of this induction period can vary considerably between systems, with reports ranging from a few seconds^[^ ^13^ ^]^ to over several hours.^[^ ^14^ ^]^ However, there is little understanding of the physical or chemical processes that underpin this induction period, so we have no way of predicting or modifying it. Given the potentially extreme implications of the induction period for determining mechanochemical kinetics, gaining control of this kinetic regime is needed before we can hope to design a mechanochemical process.

Probing how different process parameters affect mechanochemical reactions can be done through ex situ analysis of the reacting material.^[^ ^6^, ^15^ ^]^ In some cases, however, the stop‐start procedures required for ex situ analysis can lead to modified reaction pathways^[^ ^16^ ^]^ or even entirely different reaction products.^[^ ^17^ ^]^ It is not possible to predict a priori when such anomalous behavior might occur. In this respect, time‐resolved in situ (TRIS) methods have become key tools for probing the intricacies of mechanochemical transformations.^[^ ^18^, ^19^ ^]^ Most notably, with advances in the technique and data processing strategies, both phase and crystal sizes can be extracted from TRIS powder X‐ray diffraction (PXRD).^[^ ^20^ ^]^ These TRIS‐PXRD studies have lent support to ex situ studies,^[^ ^14^ ^]^ which together indicate that induction periods are associated with the mechanical activation of reagent material. However, direct evidence of this physical origin for induction periods remains elusive.

If induction periods do relate to the physical activation of reagents, we hypothesize that their length should be tuneable by pre‐activating the starting reagent powders by ball milling them separately prior to the reaction. In this study “pre‐activation” refers specifically to the mechanical milling of one solid reagent (150 mg reactant, at the conditions specified through the text and summarized in Figure 2) on its own, before being added to the main reaction mixture. This pre‐milling step deliberately modifies the powder, physically and energetically, before it is combined with the second component. This can potentially alter the subsequent reaction kinetics and outcomes.

Here, we use TRIS‐PXRD to investigate how the kinetics of mechanochemical reactions can be modulated by pre‐activating the starting reagents. For this purpose, we selected three co‐crystal systems as models: theophylline (TP) + benzamide (BA), TP + nicotinamide (NIC), and anthranilic acid (ANT) with caffeine (CAF), Scheme 1b. These co‐crystals were selected as they are known to form under neat mechanochemical conditions with measurable induction periods.

## Results and Discussion

**Figure 1:**
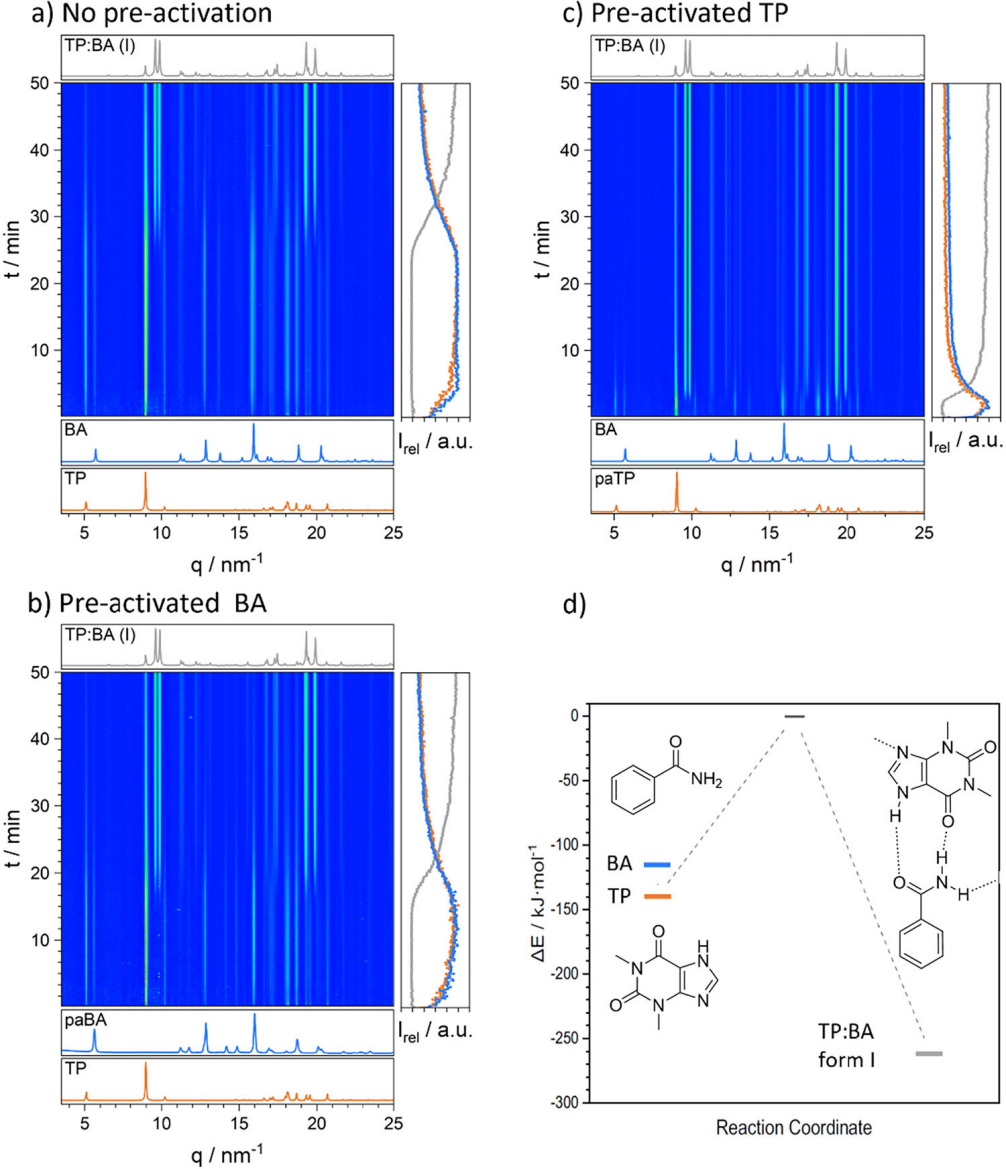
Mechano‐co‐crystallization profiles for our theophylline (TP) + benzamide (BA), monitored by TRIS PXRD. a) Reaction performed using commercial material, at 50 Hz using an 8 mm milling ball. b) Mechano‐co‐crystallization using pre‐activated BA. c) Mechano‐co‐crystallization using pre‐activated TP. d) “Reaction profile” at PBE‐D3 for the transformation from crystalline starting crystalline materials via ‘free’ gas phase molecules, to the final co‐crystal product for TB + BA.

As a baseline for the kinetics of our model mechanochemical co‐crystallizations we used TRIS‐PXRD to monitor their ball milling induced transformations (ESI S4). In each case, the transformations occurred as expected, with the reagents transforming into their respective co‐crystal products within relatively short milling times. Each mechanochemical co‐crystallization exhibited a clear induction period, ranging from *ca* 25 min for TP + BA, Figure 1a, down to only *ca* 9 min for the TP + NIC system.

With the aim to modify the reaction rates of these co‐crystallizations, we sought to identify which reagents likely contribute most to reaction induction times. To do this, we hypothesized that the rate‐limiting step corresponds to the dissociation of the most stable reagent crystal (i.e., where it is most difficult to dissociate the parent lattice to form the co‐crystal product), as revealed by density functional theory simulations, Figure 1d. At PBE‐D3 level of theory, our calculations indicate that for the TP + BA system the lattice energy (*E_latt_*) of TP is *ca* 23 kJ mol^−1^ more stable than BA. The relative reagent stabilities do not change when the same calculations are performed at the PBE + D2, PBE + D3(BJ), or PBE + D4 levels of theory (see ESI S3.1.1 and S3.1.2) lending support to the validity of our computed landscapes. We therefore propose that dissociation of TP is the rate limiting step for the TP + BA mechanochemical co‐crystallization. As such, we predict that pre‐activating TP will have the largest effect on the mechano‐co‐crystallization reaction rate.

Using powders taken directly from the suppliers (details in ESI S1.1), the mechanochemical co‐crystallization of TP + BA proceeded as expected from literature, Figure 1a.^[^ ^20^, ^21^ ^]^ When milling at 50 Hz with an 8 mm stainless steel milling ball, an induction period of 24 ± 5 min was observed. Following the induction period, reflections assigned to the co‐crystal product began to appear. Complete transformation to the stoichiometric co‐crystal was observed after 47 ± 5 min of ball milling (see ESI S4.1.1).

To assess the effects of pre‐activating the reagent powders, we ball milled a powder (150 mg) of BA for 30 min with a 10 mm stainless steel ball (4.045 ± 0.005 g) at 50 Hz (∼0.6 mJ impact^−1^). This activated powder was then mixed with a stoichiometric quantity of non‐activated TP and the mechanochemical co‐crystallization was performed, monitored by TRIS‐PXRD, Figure 1b and ESI S4.1.3). We observed no notable change in the reaction kinetics when pre‐activated BA was used in the reaction, with the induction period still requiring 21 ± 5 min, and 39 ± 3 min needed to reach complete transformation. The finding that pre‐activating BA has limited effect on the reaction rate is consistent with our calculated energy surfaces, Figure 1d.

In stark contrast, when a sample of TP was pre‐activated (ball milled for 30 min at 50 Hz with a 10 mm stainless steel ball), the mechanochemical co‐crystallization occurred much more rapidly, Figure 1c. Our TRIS‐PXRD studies showed that with pre‐activated TP, an induction period of only 3 ± 1 min was required before crystallization of the product phase began, with the reaction achieving completion after only 7 ± 2 min of ball milling. By reducing the pre‐activation milling time to 5 min, a longer induction period of 6 ± 1 min was observed (see ESI S4.1.4), indicating that the rate‐enhancement effect is sensitive to the magnitude of pre‐activation.

In the present study, TRIS monitoring revealed a direct mechanochemical conversion of the reactants into the final products under rigorously dry conditions. This implies that any transient intermediates, if they were generated, were either amorphous or had insufficient crystallinity to be detected within the temporal and spatial limits of the in situ diffraction experiment. Nevertheless, this information is important as it clarifies the observed reaction pathway under the applied conditions and highlights the importance of TRIS in distinguishing between direct transformations and mechanisms involving transient intermediates.

**Figure 2:**
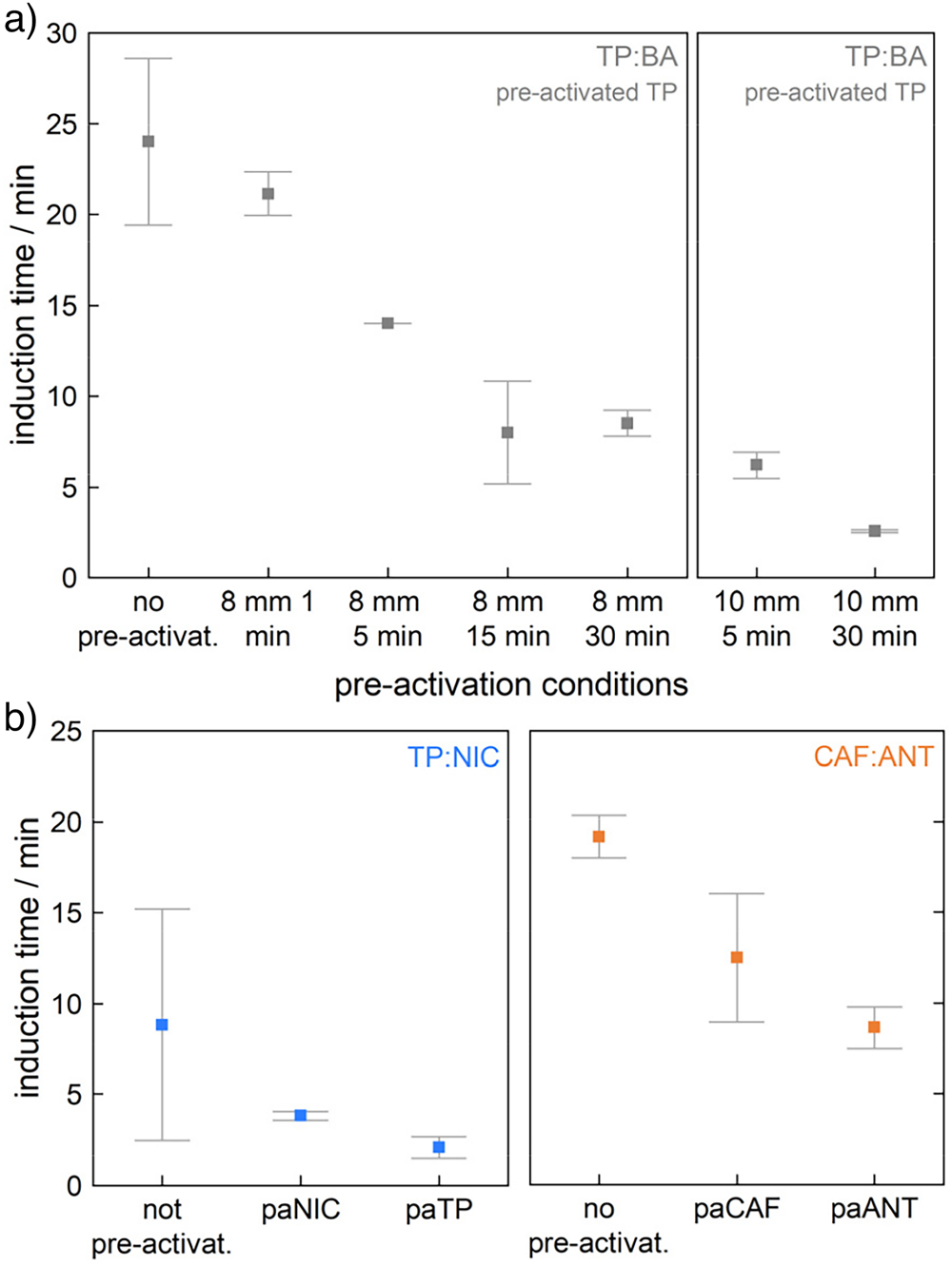
Induction time for the mechano‐co‐crystallizations. a) Induction time for the crystallization of TP + BA as a result of different pre‐activation (pa) procedures (time, ball size). b) Induction time for co‐crystallization of TP + NIC and CAF + ANT, with non‐pre‐activated and pre‐activated (details in text) reagents.

To increase our ability to resolve differences in the rate‐enhancement obtained through pre‐activation of TP, we opted for a lighter milling ball (8 mm diameter, 2.07 ± 0.005 g). In doing so, we reduced the amount of energy injected into the powder with each impact (from ∼0.6 to ∼0.4 mJ impact^−1^), with the aim to magnify the difference between 5‐ and 30‐min pre‐activation milling times. Using this lighter milling ball, we performed a series of experiments wherein a powder of TP was pre‐activated for increasing lengths of time, ranging from 1 min through to 30 min. Remarkably, TRIS‐PXRD studies (see ESI S4.1) showed a systematic decrease in the length of the induction period with increasing pre‐activation time, Figure 2a.

A slight reduction of the induction period time to 21 ± 1 min was observed after only 1 min pre‐activation, reducing further to 8 ± 3 min with 15 min pre‐activation. As we increased the pre‐activation time beyond 15 min with an 8 mm ball, we did not observe further reduction of the induction period. This indicates that we may have reached an energy threshold for the material activation that is only surmounted by further increasing the kinetic energy of milling using a heavier milling ball.^[^ ^14^ ^]^ By considering the effect that different pre‐activation times had on the overall reaction time, we find that there is in fact an optimal pre‐activation regiment (5 min with an 8 mm ball), which leads to the shortest overall reaction time, ESI Figure S4.1.10. These findings strongly support our initial hypothesis that pre‐activating the most stable reagent will have the greatest effect on the overall reaction rate. Moreover, our findings corroboate our previous ex situ results,^[^ ^14^ ^]^ indicating that activation is a cumulative phenomenon, and can be used to optimize mechanochemical reaction kinetics.

Having observed the significant impact that pre‐activation can have on the reaction rate of TP + BA, we next explored the phenomenon on a second co‐crystal involving TP, namely TP + nicotinamide (NIC). For this co‐crystallization reaction, our PBE‐D3 calculations indicated that *E_latt_* of TP was *ca* 16 kJ mol^−1^ more stable than that of NIC (see ESI S3.1.2 and S3.1.3) independent of the selection of dispersion correction. Thus, once again, our calculations indicated that the pre‐activation of TP should be expected to have the largest effect on the reaction kinetics of the co‐crystallization process.

When the co‐crystallization was performed using the non‐activated materials, an induction period of *ca* 9 ± 6 min was observed when milling at 50 Hz with an 8 mm milling ball. Under these conditions, the co‐crystallization required 82 ± 5 min ball milling to achieve a complete conversion to the co‐crystal product, Figure 2b and ESI S4.2.1. Consistent with our calculated energy profiles, the activation of TP for 30 min (10 mm milling ball at 50 Hz) reduced the induction period to only 2 ± 0.5 min, Figure 2b and ESI S4.2.2. However, the pre‐activation of NIC using the same milling parameters also caused a notable influence on the induction period, with the induction period reducing to 4 ± 0.2 min, Figure 2b and ESI S4.2.3. Both pre‐activation procedures are accompanied by a significant reduction in the overall reaction rate, decreasing to 42 ± 12 min when TP is pre‐activated and to 51 ± 1 min when NIC is pre‐activated (Figure 2b and ESI S4.2). Hence, for both of the mechano‐co‐crystallization reactions that involve TP, we see the most significant rate enhancement when the most stable starting reagent is pre‐activated.

Finally, we investigated whether this pre‐activation phenomenon holds for a chemically unrelated system: the co‐crystallization of anthranilic acid (ANT) and caffeine (CAF). In this system, our energy landscapes suggest that ANT is *ca* 23 kJ mol^−1^ more stable than CAF, thereby suggesting that the activation of ANT should have a dominant influence on the co‐crystallization kinetics.

**Figure 3:**
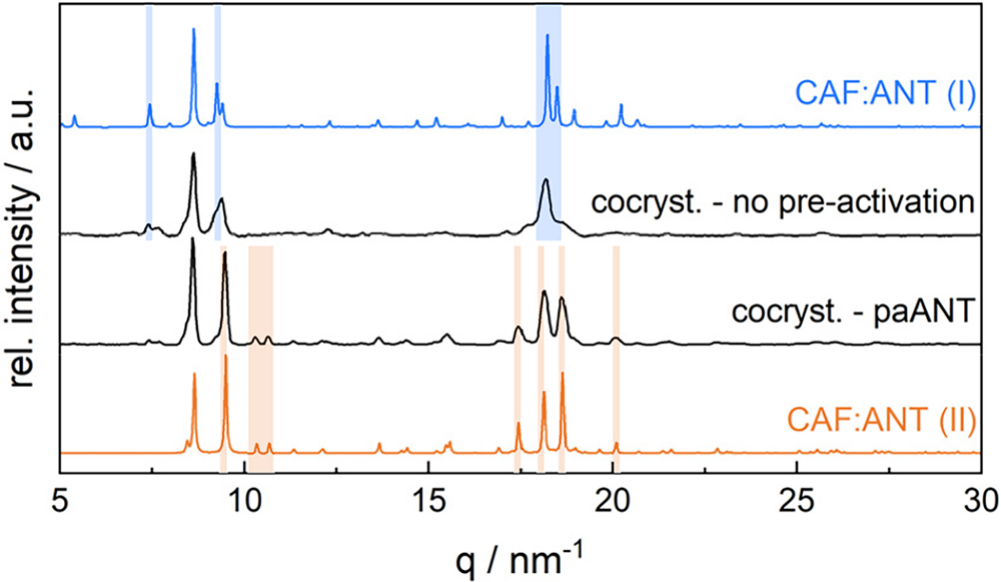
Diffraction patterns of the final product of CAF:ANT mechano‐co‐crystallization experiments, obtained by TRIS‐PXRD. We compare the outcomes using reagents with no‐pre‐activation against a reaction where ANT was pre‐activated (pa). Experimental patterns are compared with simulated PXRD profiles for CAF:ANT polymorph I (blue) and II (orange) PXRD.

Using the non‐activated material, we observed a 19 ± 1 min induction period for the mechano‐co‐crystallization of ANT + CAF when ball milling at 50 Hz with an 8 mm milling ball (Figure 2b and ESI S4.3.1). A total of 73 ± 12 min was needed under these conditions to achieve complete transformation. By pre‐activating CAF (30 min milling at 50 Hz with a 10 mm milling ball), we observed only a small influence on the reaction rate, Figure 2b and ESI S4.3.2. Under these pre‐activation conditions, the induction time decreased to 12.5 ± 3.5 min, with the reaction complete after 72.5 ± 3.5 min. A larger effect was observed when ANT was pre‐activated (30 min milling at 50 Hz with a 10 mm milling ball), leading to an induction period of 9 ± 1 min and a complete reaction time of 32 ±  5 min, Figure 2b and ESI S4.3.3. These effects are consistent with our energy diagram, again finding that the pre‐activation of the most stable phase has the largest influence on the reaction kinetics. It is worth noting that the pre‐activation of ANT did not only enhance the rate of the mechano‐co‐crystallization, but it led to the formation of a different polymorph of the co‐crystal product (Figure 3) within the milling times that we explored (up to *ca* 70 min). Ex situ tests confirm that, without pre‐milling, this second polymorph can be obtained after 3 h of milling, ESI S4.3.4. This finding serves as an important example of how sensitive mechanochemical transformations can be to the history of the starting reagents, and the critical importance of understanding and reporting the nature of the starting solid phases in any mechanochemical reaction.

In mechanochemical processes, the kinetics are governed by the ability to form heterogeneous contacts between reagent powders. In this respect, one might expect that—in addition to an energetic argument—one could link the effects of pre‐activation to an increase in powder surface area.^[^ ^7^ ^]^ To explore whether a change in surface area—as opposed to bulk activation—could be responsible for the observed kinetic enhancements in the present system, we performed a set of BET measurements on the “as supplied” and mechanically activated powders for each of our starting reagents, ESI S6.1.

For the TP + BA system, even though activating BA had no effect on the kinetics of our transformation, we observed a *ca* fourfold increase in the surface area of BA powders, in contrast to an only twofold increase in TP once activated. Similarly, activated NIC also exhibited a *ca* fourfold increase in its surface area, for ANT a 20‐fold increase was observed. One has to keep in mind that all the powders investigated remained below the level at which gas sorption analysis is unambiguous. Due to the limited mass of the samples and the sensitivity of the instruments, any differences observed in the specific surface area must be interpreted with caution and are not suitable for unambiguous quantitative discussion. Regardless our data suggest that the impact of pre‐activation extends beyond mere changes in particle size or surface area. Mechanochemical pre‐activation results in structural modifications within the bulk of the reagent crystals. Our previous studies indicated that ball milling, for example, can increase a particle's internal energy, presumably by introducing a high density of defects, strains and amorphous regions.^[^ ^14^ ^]^ Our present TRIS data suggest that this excess internal energy lowers reaction barriers and enhances the overall reaction rates.

Our results therefore suggest that the generation of high surface area—whilst certainly important for dictating mechanochemical kinetics—is not necessarily a reliable indicator of increased reaction rates. We posit that kinetic models of mechanochemical reactions should account for both energetic and physical parameters, and emphasize the effectiveness of pre‐activation in accelerating reactions and directing selectivity in mechanochemical transformations, providing a mechanistically sound approach to the rational design of solid‐state reactions.

## Conclusion

Mechanochemical transformations exhibit remarkably unique kinetics, which contrast conventional profiles seen in solution chemistry. This means that common approaches to manipulate and control these reaction kinetics will not hold and new understanding of the mechanisms giving rise to mechanochemical kinetics are needed. Using time‐resolved in situ (TRIS) powder X‐ray diffraction (PXRD) we here investigate how pre‐activation of the reacting powders affect the reaction kinetics of mechanochemical co‐crystallization. We studied three systems: TP + BA, TP + NIC, and CAF + ANT. Based on DFT simulations of the energetic landscapes of co‐crystallization we posited that activation of the most stable reaction component (TP and ANT) should have the largest effect on the reaction rates.

For all three of our mechano‐co‐crystallizations, we demonstrate how pre‐activation of the most stable starting reagent has the biggest impact on the subsequent reaction rates. Moreover, our study suggests that pre‐activation is a cumulative effect, with longer and more energetically intense pre‐activation protocol leading to more significant rate enhancements. Although nitrogen sorption measurements were undertaken, the absolute surface areas of all the powders investigated remained below the level at which gas sorption analysis is unambiguous. Any differences observed in the specific surface area must therefore be interpreted with caution. However, our studies do suggest that kinetic enhancements that arise from mechanical activation are most likely associated with mechanically induced structural modifications (e.g., defect formation), as opposed to being dominated by an increase in particle surface area. This is consistent with previous ex situ studies of pre‐activation. In addition to the rate‐enhancement effects of pre‐activation, we also find for ANT + CAF that pre‐activating reagent powders can which polymorphic form is obtained from the mechano‐co‐crystallization.

Many different types of mechanochemical reactions exhibit marked induction periods, including organic syntheses, co‐crystallizations, the synthesis of metal–organic frameworks, and inorganic metathesis reactions. Whilst our study focuses on a limited number of exemplary co‐crystallizations, we fully anticipate that the concepts will be widespread for any system that exhibits a measurable induction period. Overall, our study finds that mechanochemical reaction profiles—both kinetics and the resulting product phase—are sensitive to the activation state of the starting material. Our results therefore offer strong indication that pre‐activation offers a largely unexplored route to manipulate mechanochemical processes. Our results also serve as an important example of the need to understand the history of the starting solid materials if one is to fully understand and control a mechanochemical transformation.

## Supporting Information

The authors have cited additional references within the Supporting Information.^[^ ^22^, ^23^, ^24^, ^25^, ^26^, ^27^, ^28^, ^29^, ^30^, ^31^, ^32^, ^33^, ^34^, ^35^, ^36^, ^37^, ^38^, ^39^, ^40^, ^41^, ^42^ ^]^

## Conflict of Interests

The authors declare no conflict of interest.

## Supporting information



Supporting Information

## Data Availability

The data that support the findings of this study are available from the corresponding author upon reasonable request.
