# Supplementary material for: Pre‐Activation as a Route for Tuning the Kinetics of Mechanochemical Transformations
Source: Angew Chem Int Ed Engl. 2025 Nov 23;65(3):e16632. doi: 10.1002/anie.202516632 (PMC12811646; doi:10.1002/anie.202516632)
Supplement: Supplementary file 1 — Supporting Information [file ANIE-65-e16632-s001.docx]

**ELECTRONIC SUPPLEMENTARY INFORMATION**

Pre-activation as a route for tuning the kinetics of mechanochemical transformations

Christian Heinekamp,^[+,a,b]^ Tahlia M. Palmer,^[+,c]^ Dominik Al-Sabbagh,^[a]^ Carsten Prinz,^[a]^ Stefan Michalik,^[d]^ Adam A. L. Michalchuk,*^[a,c]^ and Franziska Emmerling*^[a,b]^

^[a] Federal Institute for Materials Research and Testing (BAM), Richard-Wilstaetter Str 11, 12489, Berlin Germany^

^[b] Department of Chemistry, Humboldt University, 12489, Berlin Germany^

^[c] School of Chemistry, University of Birmingham, Edgbaston, UK, B15 2TT^

^[d] Diamond Light Source Ltd., Harwell Science and Innovation Campus, Didcot, Oxfordshire OX11 0DE, UK^

**CONTENTS**

S1 Experimental Methods…………………………………………………………………………………………………….... S1

S2 Computational Details ………………………………………………………………………………………………………. S3

S3 Crystal Lattice Energy and Cocrystal Energy Landscapes …………………………………………………… S7

S4 Time Resolved *In Situ* (TRIS) Powder X-ray Diffraction (PXRD) …….……………………………………. S11

S5 Scanning Electron Microscopy Analysis ……………………………………………………………………………. S20

S6 Brunauer-Emmett-Teller (BET) Analysis …………………………………………………………………………… S24

S7 References ………………………………………………………………………………………………………………………. S25

**S1 Experimental Details**

**S1.1 Materials.**

The chemicals (nicotinamide–SIGMA, 99%; benzamide–SIGMA, 99%; theophylline–ACROS, 99%; caffeine–Acros Organics, 98.5%; anthranilic acid–Acros Organics, ≥ 98%) were obtained commercially and used without further purification.

**S1.2 Time-resolved *in situ* (TRIS) powder X-ray diffraction (PXRD)**

TRIS PXRD measurements were performed at the I12 JEEP beamline^[1]^ of the Diamond Light Source for TP:BA and at the µSpot beamline^[2]^ of the BESSY II synchrotron (Helmholtz-Zentrum Berlin für Material und Energie), for CAF:ANT and TP:NIC. At Diamond Light source a monochromatic beam of 52.954 keV with beam size of 100 µm x∙100 µm was used for diffraction, with an acquisition time of 10 s, aligned to the edge of the jar as described in our previous work.^[3]^ At BESSY II a monochromatic beam of 17.0 keV with a beam size of 150 µm was used, with acquisition time of 10 s and 8 s of exposure. Ball milling was performed using a Fritsch Pulverisette P23 vibratory ball mill with custom designed jars (details elsewhere^[3]^) with X-ray transparent polymethylmethacrylate (PMMA) windows. The incident beam was aligned to the tangent of the jar to minimize aberration to the Bragg reflections. Ball milling cocrystallization was performed at 50 Hz with a single 8 mm stainless steel ball (2.07 g). Pre-activation (*i.e.* pre-milling) was performed at various frequencies (see in text) with a single stainless steel milling ball (size in text). TRIS-PXRD data of the Diamond Light Source were integrated using the Dawn software suite.^[4]^ TRIS-PXRD data from BESSY II were processed using the DPDAK software suite. Background correction was performed with the public python code BackgroundCorrectionUI at <https://github.com/embienert/BackgroundCorrectionUI>. The starting and ending times of the reactions were determined manually.

**S1.3 Scanning electron microscopy (SEM)**

The scanning electron microscopy (SEM) analysis was performed on a XL 30 ESEM equipped with a tungsten cathode (FEI, Eindhoven, in 2020 electronic upgrade by point electronic GmbH) operating with 20 keV acceleration voltage and using a secondary electron (SE) detector. The samples were vapour-deposited with 30 nm gold prior the measurement for electronic conductivity.

**S1.4 N_2_ adsorption/ Brunauer Emmett Teller analysis (BET)**

Nitrogen adsorption/desorption isotherms at -196 °C (77 K) were measured with a Micromeritics ASAP 2020 V4.04 surface area and porosity analyzer. Prior to measurements, samples were degassed at room temperature and 200 ̊C for 24 h in vacuum. No further pre-treatment was applied to exclude interference with milling activation. Specific surface areas were calculated using the Brunauer-Emmett-Teller (BET) method^[5,6]^ with a molecular sectional area of 0.162 nm² according to ISO 9277 (2010).^[7]^

**S1.5 Ideal Impact Energies**

To approximate the impact energies, we assume ideal linear impact geometry. Noting the milling jar translates 8 mm per oscillation, we assume a ball-to-wall impact occurs out-of-phase, such that the impact energy is approximately twice the kinetic energy of the milling ball itself, Table X.

| **Milling frequency** | **Ball size and mass** | **Approx. Impact Energy** |
| --- | --- | --- |
| 50 Hz (0.02 s^-1^) | 10 mm (4.045 g) | 0.6 mJ |
| 50 Hz (0.02 s^-1^) | 8 mm (2.07 g) | 0.4 mJ |

**S2 COMPUTATIONAL DETAILS**

**S2.1 Computational approach.**

All calculations were performed using plane wave density functional theory (DFT) as implemented in CASTEP v24.1.^[8]^ Calculations were done using the Generalized Gradient Approximation of Perdew-Burke-Ernzerhof (PBE),^[9]^ with variations of Grimme’s semi-empirical dispersion correction (D2,^[10]^ D3,^[11]^ D3BJ,^[12]^ D4^[13]^). The wavefunction was expanded in plane waves to a kinetic energy cut-off of 1100 eV, and the core potential was approximated with norm-conserving pseudopotentials generated on the fly in CASTEP v24.1. The electronic structure was sampled on a Monkhorst-Pack grid of points, with spacing 0.08 Å^-1^. The wavefunction was accepted as being converged once the electronic energy <1×10^−10^ eV and the electronic eigenvalue <1×10^−12^ eV.

Input geometries for each system were taken from the Cambridge Crystallography Database (CCDC): theophylline (tp, REF: BAPLOT01^[14]^), benzamide (ba, REF: BZAMID02^[15]^), tp:ba (REF: RABXIE02^[16]^), anthranilic acid (ant, REF: AMBACO06^[17]^), caffeine (caff, REF: NIWFEE03^[18]^) ant:caff form 1 (REF: ZOBCOK^[19]^), ant:caff form 2 (REF: ZOBCOK01^[19]^), nicotinamide (nic, REF: NICOAM06^[20]^), and tp:nic (REF: EVEYAH^[21]^).

**S2.2 Computational Parameters.**

Full geometry optimization was performed for each system using the computational methods outlined in Section S2.1. The geometry relaxation was accepted once the following convergence criteria were achieved: SCF convergence < 1x10^-10^ eV, total energy <3×10^−7^ eV atom^−1^, forces <1×10^−4^ eV Å^−1^ and the maximum stress <0.01 GPa, using the Limited-memory Broyden-Fletcher-Goldfarb-Shannon (LBFGS) algorithm. The Fourier grids were sampled using scaling factors of 2.0.The geometries resulting from each of these calculations are reported in Tables S2.1-S2. The structure of anhydrous caffeine contains significant static disorder. To perform lattice energy calculations, we extracted a single orientation of the caffeine molecules, from which the lattice energy was calculated. We note that this is a significant approximation and likely to underestimate the lattice energy of caffeine. However, we anticipate the underestimation to be minimal, given these values provide total cocrystal formation enthalpies on the order of magnitude expected for organic cocrystals (see Table S3.10)

**Table S2.2.1.** Comparing the unit cell parameters for benzamide obtained from experiment (exp, T=15 K) and from DFT calculations at PBE level using different dispersion corrections. Differences are calculated as $\Delta=100(\mathrm{calc}-\exp)/\exp$ .

|  | **a / Å** | **b / Å** | **c / Å** | **α /^◦^** | **β /^◦^** | **γ /^◦^** | **Vol / Å^3^** |
| --- | --- | --- | --- | --- | --- | --- | --- |
| Exp | 5.529 | 5.033 | 21.343 | 90.000 | 88.730 | 90.00 | 593.776 |
| D2 | 5.482 | 5.002 | 20.409 | 90.000 | 87.057 | 90.000 | 558.880 |
| Δ^D2^% | -0.85% | -0.62% | -4.38% | - | -1.89% | 0.00% | -5.88% |
| D3 | 5.559 | 5.008 | 21.568 | 90.000 | 89.328 | 90.000 | 600.367 |
| Δ^D3^ % | 0.54% | -0.50% | 1.06% | - | 0.67% | - | 1.11% |
| D3BJ | 5.564 | 5.015 | 21.239 | 90.000 | 89.043 | 90.000 | 592.584 |
| Δ^D3BJ^ % | 0.63% | -0.35% | -0.49% | - | 0.35% | - | -0.20% |
| D4 | 5.584 | 5.026 | 21.357 | 90.000 | 89.251 | 90.000 | 599.294 |
| Δ^D4^ % | 0.99% | -0.14% | 0.07% | - | 0.59% | - | 0.93% |

**Table S2.2.2** Comparing the unit cell parameters for theophylline obtained from experiment (exp, T=293 K) and from DFT calculations at PBE level using different dispersion corrections. Differences are calculated as $\Delta=100(\mathrm{calc}-\exp)/\exp$ .

|  | **a / Å** | **b / Å** | **c / Å** | **α /^◦^** | **β /^◦^** | **γ /^◦^** | **Vol / Å^3^** |
| --- | --- | --- | --- | --- | --- | --- | --- |
| Exp | 24.612 | 3.830 | 8.501 | 90.000 | 90.000 | 90.000 | 801.380 |
| D2 | 24.228 | 3.652 | 8.513 | 90.000 | 90.000 | 90.000 | 753.201 |
| Δ^D2^% | -1.56% | -4.66% | 0.14% | - | - | - | -6.01% |
| D3 | 24.001 | 3.912 | 8.455 | 90.000 | 90.000 | 90.000 | 793.898 |
| Δ^D3^ % | -2.48% | 2.14% | -0.54% | - | - | - | -0.93% |
| D3BJ | 24.070 | 3.865 | 8.489 | 90.000 | 90.000 | 90.000 | 789.767 |
| Δ^D3BJ^ % | -2.20% | 0.91% | -0.14% | - | - | - | -1.45% |
| D4 | 24.169 | 3.835 | 8.519 | 90.000 | 90.000 | 90.000 | 789.477 |
| Δ^D4^ % | -1.80% | 0.11% | 0.21% | - | - | - | -1.49% |

**Table S2.2.3.** Comparing the unit cell parameters for nicotinamide obtained from experiment (exp, T=90 K) and from DFT calculations at PBE level using different dispersion corrections. Differences are calculated as

$\Delta=100(\mathrm{calc}-\exp)/\exp$ .

|  | **a / Å** | **b / Å** | **c / Å** | **α /^◦^** | **β /^◦^** | **γ /^◦^** | **Vol / Å^3^** |
| --- | --- | --- | --- | --- | --- | --- | --- |
| Exp | 3.856 | 15.630 | 9.370 | 90.000 | 98.188 | 90.000 | 559.029 |
| D2 | 3.748 | 15.462 | 9.245 | 90.000 | 96.795 | 90.000 | 531.958 |
| Δ^D2^% | -2.82% | -1.08% | -1.33% | - | -1.42% | - | -4.84% |
| D3 | 4.075 | 15.196 | 9.243 | 90.000 | 97.235 | 90.000 | 567.754 |
| Δ^D3^ % | 5.66% | -2.78% | -1.36% | - | -0.97% | - | 1.56% |
| D3BJ | 3.929 | 15.528 | 9.254 | 90.000 | 97.671 | 90.000 | 559.469 |
| Δ^D3BJ^ % | 1.88% | -0.65% | -1.24% | - | -0.53% | - | 0.08% |
| D4 | 3.914 | 15.697 | 9.258 | 90.000 | 97.768 | 90.000 | 90.000 |
| Δ^D4^ % | 1.50% | 0.43% | -1.19% | - | -0.43% | - | -83.90% |

**Table S2.2.4.** Comparing the unit cell parameters for anthranilic acid obtained from experiment (exp, T=295 K) and from DFT calculations at PBE level using different dispersion corrections. Differences are calculated as

$\Delta=100(\mathrm{calc}-\exp)/\exp$ .

|  | **a / Å** | **b / Å** | **c / Å** | **α /^◦^** | **β /^◦^** | **γ /^◦^** | **Vol / Å^3^** |
| --- | --- | --- | --- | --- | --- | --- | --- |
| Exp | 12.226 | 15.336 | 7.560 | 90.000 | 152.560 | 90.000 | 653.204 |
| D2 | 11.969 | 14.951 | 7.399 | 90.000 | 152.789 | 90.000 | 605.452 |
| Δ^D2^% | -2.10% | -2.51% | -2.13% | - | 0.15% | - | -7.31% |
| D3 | 12.082 | 15.427 | 7.494 | 90.000 | 152.770 | 90.000 | 639.089 |
| Δ^D3^ % | -1.18% | 0.59% | -0.87% | - | 0.14% | - | -2.16% |
| D3BJ | 12.099 | 15.329 | 7.499 | 90.000 | 152.874 | 90.000 | 634.169 |
| Δ^D3BJ^ % | -1.04% | -0.04% | -0.80% | - | 0.21% | - | -2.91% |
| D4 | 12.194 | 15.308 | 7.533 | 90.000 | 152.938 | 90.000 | 639.745 |
| Δ^D4^ % | -0.26% | -0.19% | -0.35% | - | 0.25% | - | -2.06% |

**Table S2.2.5.** Comparing the unit cell parameters for caffeine obtained from experiment (exp, T=295 K) and from DFT calculations at PBE level using different dispersion corrections. Differences are calculated as $\Delta=100(\mathrm{calc}-\exp)/\exp$ .

|  | **a / Å** | **b / Å** | **c / Å** | **α /^◦^** | **β /^◦^** | **γ /^◦^** | **Vol / Å^3^** |
| --- | --- | --- | --- | --- | --- | --- | --- |
| Exp | 43.039 | 15.068 | 6.953 | 90.000 | 99.027 | 90.000 | 4453.210 |
| D2 | 43.800 | 14.748 | 6.593 | 90.000 | 107.785 | 90.000 | 4054.915 |
| Δ^D2^% | 1.77% | -2.12% | -5.19% | - | 8.84% | - | -8.94% |
| D3 | 44.090 | 14.852 | 6.962 | 90.000 | 106.347 | 90.000 | 4374.764 |
| Δ^D3^ % | 2.44% | -1.43% | 0.13% | - | 7.39% | - | -1.76% |
| D3BJ | 44.137 | 14.889 | 6.906 | 90.016 | 106.804 | 90.006 | 4344.751 |
| Δ^D3BJ^ % | 2.55% | -1.18% | -0.68% | 0.02% | 7.85% | 0.01% | -2.44% |
| D4 | 44.357 | 14.946 | 6.864 | 90.084 | 106.574 | 89.953 | 4361.373 |
| Δ^D4^ % | 3.06% | -0.81% | -1.29% | 0.09% | 7.62% | -0.05% | -2.06% |

**Table S2.2.6.** Comparing the unit cell parameters for theophylline-benzamide cocrystal obtained from experiment (exp, T=293 K) and from DFT calculations at PBE level using different dispersion corrections. Differences are calculated as Δ=100(calc−exp)/exp Δ=100(calc−exp)/exp

|  | **a / Å** | **b / Å** | **c / Å** | **α /^◦^** | **β /^◦^** | **γ /^◦^** | **Vol / Å^3^** |
| --- | --- | --- | --- | --- | --- | --- | --- |
| Exp | 10.281 | 10.281 | 26.159 | 90.000 | 90.000 | 90.000 | 2765.188 |
| D2 | 9.967 | 9.967 | 25.791 | 90.000 | 90.000 | 90.000 | 2561.836 |
| Δ^D2^% | -3.06% | -3.06% | -1.41% | - | - | - | -7.35% |
| D3 | 10.213 | 10.213 | 25.971 | 90.000 | 90.000 | 90.000 | 2708.673 |
| Δ^D3^ % | -0.67% | -0.67% | -0.72% | - | - | - | -2.04% |
| D3BJ | 10.184 | 10.184 | 25.956 | 90.000 | 90.000 | 90.000 | 2691.960 |
| Δ^D3BJ^ % | -0.95% | -0.95% | -0.78% | - | - | - | -2.65% |
| D4 | 10.184 | 10.184 | 25.956 | 90.000 | 90.000 | 90.000 | 2691.960 |
| Δ^D4^ % | -0.95% | -0.95% | -0.78% | - | - | - | -2.65% |

**Table S2.2.7.** Comparing the unit cell parameters for theophylline-nicotinamide cocrystal obtained from experiment (exp, T=293 K) and from DFT calculations at PBE level using different dispersion corrections. Differences are calculated as $\Delta=100(\mathrm{calc}-\exp)/\exp$

|  | **a / Å** | **b / Å** | **c / Å** | **α /^◦^** | **β /^◦^** | **γ /^◦^** | **Vol / Å^3^** |
| --- | --- | --- | --- | --- | --- | --- | --- |
| Exp | 3.828 | 16.604 | 21.530 | 90.000 | 99.738 | 90.000 | 1348.630 |
| D2 | 3.670 | 16.497 | 21.167 | 90.000 | 102.596 | 90.000 | 1250.588 |
| Δ^D2^% | -4.13% | -0.64% | -1.69% | - | 2.87% | - | -7.27% |
| D3 | 3.832 | 16.597 | 21.369 | 90.000 | 100.315 | 90.000 | 1337.068 |
| Δ^D3^ % | 0.11% | -0.04% | -0.75% | - | 0.58% | - | -0.86% |
| D3BJ | 3.788 | 16.574 | 21.438 | 90.000 | 99.717 | 90.000 | 1326.470 |
| Δ^D3BJ^ % | -1.05% | -0.18% | -0.43% | - | -0.02% | - | -1.64% |
| D4 | 3.773 | 16.596 | 21.539 | 90.000 | 99.433 | 90.000 | 1330.335 |
| Δ^D4^ % | -1.44% | -0.05% | 0.04% | - | -0.31% | - | -1.36% |

**Table S2.2.8.** Comparing the unit cell parameters for anthranilic acid-caffeine form 1 cocrystal obtained from experiment (exp, T=298 K) and from DFT calculations at PBE level using different dispersion corrections. Differences are calculated as $\Delta=100(\mathrm{calc}-\exp)/\exp$

|  | **a / Å** | **b / Å** | **c / Å** | **α /^◦^** | **β /^◦^** | **γ /^◦^** | **Vol / Å^3^** |
| --- | --- | --- | --- | --- | --- | --- | --- |
| Exp | 8.485 | 13.424 | 14.886 | 111.725 | 92.846 | 93.528 | 1567.350 |
| D2 | 8.314 | 13.171 | 14.198 | 111.470 | 94.334 | 94.804 | 1432.233 |
| Δ^D2^% | -2.02% | -1.88% | -4.62% | -0.23% | 1.60% | 1.36% | -8.62% |
| D3 | 8.420 | 13.293 | 14.810 | 110.990 | 92.926 | 94.639 | 1536.882 |
| Δ^D3^ % | -0.77% | -0.98% | -0.51% | -0.66% | 0.09% | 1.19% | -1.94% |
| D3BJ | 8.447 | 13.267 | 14.724 | 111.251 | 93.340 | 94.415 | 1526.656 |
| Δ^D3BJ^ % | -0.46% | -1.17% | -1.08% | -0.42% | 0.53% | 0.95% | -2.60% |
| D4 | 8.515 | 13.279 | 14.702 | 111.721 | 93.415 | 94.112 | 1533.805 |
| Δ^D4^ % | 0.35% | -1.08% | -1.23% | 0.00% | 0.61% | 0.63% | -2.14% |

**Table S2.2.9.** Comparing the unit cell parameters for anthranilic acid-caffeine form 2 cocrystal obtained from experiment (exp, T=180 K) and from DFT calculations at PBE level using different dispersion corrections. Differences are calculated as $\Delta=100(\mathrm{calc}-\exp)/\exp$

|  | **a / Å** | **b / Å** | **c / Å** | **α /^◦^** | **β /^◦^** | **γ /^◦^** | **Vol / Å^3^** |
| --- | --- | --- | --- | --- | --- | --- | --- |
| Exp | 14.538 | 11.079 | 10.025 | 90.000 | 90.000 | 90.000 | 1614.660 |
| D2 | 14.095 | 10.683 | 9.902 | 90.000 | 90.000 | 90.000 | 1491.105 |
| Δ^D2^% | -3.05% | -3.58% | -1.22% | - | -- |  | -7.65% |
| D3 | 14.186 | 11.175 | 10.016 | 90.000 | 90.000 | 90.000 | 1587.800 |
| Δ^D3^ % | -2.42% | 0.86% | -0.09% | - | - | - | -1.66% |
| D3BJ | 14.193 | 11.075 | 10.062 | 90.000 | 90.000 | 90.000 | 1581.631 |
| Δ^D3BJ^ % | -2.37% | -0.04% | 0.38% | - | - | - | -2.05% |
| D4 | 14.300 | 11.010 | 10.082 | 90.000 | 90.000 | 90.000 | 1587.299 |
| Δ^D4^ % | -1.64% | -0.63% | 0.58% | - | -- |  | -1.69% |

**S3 CRYSTAL LATTICE ENERGY AND COCRYSTAL ENERGY LANDSCAPES**

**S3.1 Cocrystal Lattice Energies.**

Following optimization, the symmetry independent molecule was extracted and optimized in a box of 20 Å x 20 Å x 20 Å to simulate the electronic energy of the gas phase molecule, $E_{mol}$.

The total lattice energy ($E_{latt}$) for each system with *z* asymmetric units in the unit cell was then calculated according to

$$E_{latt}=\frac{E_{tot}}{z}-\sum_{i} E_{mol_{i}}$$

where *i* runs over the molecules in the asymmetric unit and $E_{tot}$ is the total electronic internal energy calculated for the unit cell. The values for $E_{latt}$ calculated at the various computational levels are reported in Tables S3.1-S3.

**Table S3.1.1.** The total internal and lattice energies for benzamide (z=4) from DFT calculations at PBE level using different dispersion corrections.

|  | $\boldsymbol{E}_{\boldsymbol{tot}}$ **/kJ.unit cell^-1^** | **E_latt_ /kJ mol^-1^** |
| --- | --- | --- |
| D2 | -7826.053 | -115.171 |
| D2_mol_ | -1955.320 |  |
| D3 | -7825.810 | -119.758 |
| D3_mol_ | -1955.211 |  |
| D3BJ | -7826.825 | -116.554 |
| D3BJ_mol_ | -1955.498 |  |
| D4 | -7826.663 | -112.818 |
| D4_mol_ | -1955.497 |  |

**Table S3.1.2.** The total internal and lattice energies for theophylline (z=4) from DFT calculations at PBE level using different dispersion corrections.

|  | $\boldsymbol{E}_{\boldsymbol{tot}}$ **/kJ.unit cell^-1^** | **E_latt_ /kJ mol^-1^** |
| --- | --- | --- |
| D2 | -12943.610 | -142.968 |
| D2_mol_ | -3234.421 |  |
| D3 | -12942.997 | -142.463 |
| D3_mol_ | -3234.273 |  |
| D3BJ | -12944.273 | -137.925 |
| D3BJ_mol_ | -3234.639 |  |
| D4 | -12944.504 | -138.641 |
| D4_mol_ | -3234.689 |  |

**Table S3.1.3.** The total internal and lattice energies for nicotinamide (z=4) from DFT calculations at PBE level using different dispersion corrections.

|  | $\boldsymbol{E}_{\boldsymbol{tot}}$ **/kJ.unit cell^-1^** | **E_latt_ /kJ mol^-1^** |
| --- | --- | --- |
| D2 | -8216.090 | -123.819 |
| D2_mol_ | -2052.739 |  |
| D3 | -8215.816 | -126.174 |
| D3_mol_ | -2052.646 |  |
| D3BJ | -8216.788 | -123.708 |
| D3BJ_mol_ | -2052.915 |  |
| D4 | -8216.686 | -121.279 |
| D4_mol_ | -2052.914 |  |

**Table S3.1.4.** The total internal and lattice energies for anthranilic acid (z=4) from DFT calculations at PBE level using different dispersion corrections.

|  | $\boldsymbol{E}_{\boldsymbol{tot}}$**/kJ.unit cell^-1^** | **E_latt_ /kJ mol^-1^** |
| --- | --- | --- |
| D2 | -9572.147 | -113.323 |
| D2_mol_ | -2391.862 |  |
| D3 | -9571.858 | -116.800 |
| D3_mol_ | -2391.754 |  |
| D3BJ | -9572.924 | -112.756 |
| D3BJ_mol_ | -2392.062 |  |
| D4 | -9572.836 | -109.936 |
| D4_mol_ | -2392.070 |  |

**Table S3.1.5.** The total internal and lattice energies for caffeine (z=20) from DFT calculations at PBE level using different dispersion corrections.

|  | $\boldsymbol{E}_{\boldsymbol{tot}}$ **/kJ.unit cell^-1^** | **E_latt_ /kJ mol^-1^** |
| --- | --- | --- |
| D2 | -68591.910 | -96.599 |
| D2_mol_ | -3428.594 |  |
| D3 | -68587.184 | -93.289 |
| D3_mol_ | -3428.392 |  |
| D3BJ | -68594.039 | -88.849 |
| D3BJ_mol_ | -3428.781 |  |
| D4 | -68595.077 | -88.159 |
| D4_mol_ | -3428.840 |  |

**Table S3.1.6.** The total internal and lattice energies for theophylline-benzamide (z=8) from DFT calculations at PBE level using different dispersion corrections. Note that values for the isolated molecular energies were taken from the appropriate calculations in Tables S3.1 & S3.2.

|  | $\boldsymbol{E}_{\boldsymbol{tot}}$ **/kJ.unit cell^-1^** | **E_latt_ /kJ mol^-1^** |
| --- | --- | --- |
| D2 | -41539.685 | -262.451 |
| D3 | -41537.674 | -262.954 |
| D3BJ | -41542.311 | -255.859 |
| D4 | -41542.502 | -253.459 |

**Table S3.1.7.** The total internal and lattice energies for theophylline-nicotinamide cocrystal (z=4) from DFT calculations at PBE level using different dispersion corrections. Note that values for the isolated molecular energies were taken from the appropriate calculations in Tables S3.2 & S3.3.

|  | $\boldsymbol{E}_{\boldsymbol{tot}}$ **/kJ.unit cell^-1^** | **E_latt_ /kJ mol^-1^** |
| --- | --- | --- |
| D2 | -21159.737 | -267.675 |
| D3 | -21158.840 | -269.295 |
| D3BJ | -21161.052 | -261.418 |
| D4 | -21161.184 | -259.755 |

**Table S3.1.8.** The internal and lattice energies for anthranilic acid-caffeine form 1 cocrystal (z=4) from DFT calculations at PBE level using different dispersion corrections. Note that values for the isolated molecular energies were taken from the appropriate calculations in Tables S3.4 & S3.5

|  | $\boldsymbol{E}_{\boldsymbol{tot}}$ **/kJ.unit cell^-1^** | **E_latt_ /kJ mol^-1^** |
| --- | --- | --- |
| D2 | -23290.834 | -210.009 |
| D3 | -23289.527 | -214.972 |
| D3BJ | -23292.016 | -207.933 |
| D4 | -23292.179 | -205.056 |

**Table S3.1.9.** The internal and lattice energies for anthranilic acid-caffeine form 2 cocrystal (z=4) from DFT calculations at PBE level using different dispersion corrections. Note that values for the isolated molecular energies were taken from the appropriate calculations in Tables S3.4 & S3.5

|  | $\boldsymbol{E}_{\boldsymbol{tot}}$ **/kJ.unit cell^-1^** | **E_latt_ /kJ mol^-1^** |
| --- | --- | --- |
| D2 | -23290.533 | -210.009 |
| D3 | -23289.497 | -214.972 |
| D3BJ | -23291.994 | -207.933 |
| D4 | -23292.140 | -205.056 |

Based on the relative crystal stabilities in Tables S3.1-S3.9, the cocrystal formation energies are all predicted to be stable, Table S3.10. Formation energies are calculated from the difference in energy between the cocrystal ($E_{cocryst}$) and the parent crystals, wherein the cocrystal comprises *n* moles of parent 1 (p1; energy $E_{p1}$) and *m* moles of parent 2 (p2; energy $E_{p2}$),

$$E_{form}=E_{cocryst}-nE_{p1}-mE_{p2}$$

**Table S3.1.10.** Formation energies (in kJ.mol^-1^) for cocrystals of theophylline + benzamide (tp:ba), theophylline + nicotinamide (tp:nic), anthranilic acid + caffeine (ant:caff) form 1 and form 2.

|  | **tp:ba** | **tp:nic** | **ant:caff Form 1** | **ant:caff Form 2** |
| --- | --- | --- | --- | --- |
| D2 | -4.312 | -0.889 | -0.086 | -0.086 |
| D3 | -0.733 | -0.657 | -4.883 | -4.883 |
| D3BJ | -1.381 | 0.215 | -6.328 | -6.328 |
| D4 | -2.000 | 0.165 | -6.961 | -6.961 |

**S3.2 Energy Profiles for Cocrystal Formation**

We assume that the formation of a cocrystal requires the complete dissociation of the molecules from the parent crystal structures, followed by nucleation and growth of the cocrystal product. Correspondingly, we can approximate a ‘reaction profile’ based on the relative lattice energies $E_{latt}$ of the starting reagents and that of the cocrystal product, Figure S3.1.

Despite minor changes in the absolute stability of the reagents and cocrystals, depending on the choice of dispersion correction, the reaction profile is conserved in all cases. For the TP+BA system, TP is consistently more stable than BA. In the TP+NIC system, TP is also most stable, in the ANT + CAF system, ANT is consistently the most stable conformer.

**Figure S3.2.1** Reaction profiles for the cocrystallisations studied in this work. Reaction profiles are shown for calculations at PBE level of theory using various dispersion corrections.

**S4 Time Resolved *In Situ* (TRIS) Powder X-ray Diffraction (PXRD)**

To monitor the rates of cocrystal formation by vibratory ball milling, we monitored the cocrystallisation for the three systems using time-resolved in situ (TRIS) powder X-ray diffraction, making us of a new TRIS set-up at the Diamond Light Source I12 beamline.

**S4.1 TRIS-PXRD of TP + BA**

To obtain a semi-quantitative description of the transformation rates of the TP + BA system under ball milling conditions, we performed a single peak integration of the powder diffraction data. This integration was done using Bragg reflections that were unique to the corresponding phase and isolated from other Bragg reflections. This corresponds to a Bragg reflection at $q\approx$5 nm^-1^ for TP, $q\approx$16 nm^-1^ for BA, and $q\approx$ 20 nm^-1^ for form I of the TP:BA cocrystal.

Ball milling reaction rates were performed using non-activated and pre-activated reagents at 50 Hz using either a 10 mm or 8 mm milling ball. Corresponding datasets are provided in Figures S4.1.1-S4.1.9, showing duplicate or triplicate experiments as available. A summary of the major rate events is given in Table S4.1.1.

**Figure S4.1.1** Thrice repeated time-resolved *in situ* (TRIS) PXRD measurements of the mechanochemical reaction with no pre-milling. In each dataset, the 1D profile (right hand side) indicate the phases present in each step (BA: orange, TP: grey, and TP:BA: purple). PXRD patterns from the starting materials and TP:BA are given for comparison.

**Figure S4.1.2** Twice repeated time-resolved *in situ* (TRIS) PXRD measurements of the mechanochemical reaction with TP pre-milled (50 Hz, 30 min, 10 mm). The right traces indicate the phases present in each step (BA: orange, TP: grey, and TP:BA: purple). PXRD patterns from the starting materials and TP:BA were given for comparison.

 **Figure S4.1.3** Thrice repeated time-resolved *in situ* (TRIS) PXRD measurements of the mechanochemical reaction with BA pre-milled (50 Hz, 30 min, 10 mm). The right traces indicate the phases present in each step (BA: orange, TP: grey, and TP:BA: purple). PXRD patterns from the starting materials and TP:BA were given for comparison.

**Figure S4.1.4** Twice repeated time-resolved *in situ* (TRIS) PXRD measurements of the mechanochemical reaction with TP pre-milled (50 Hz, 5 min, 10 mm). The right traces indicate the phases present in each step (BA: orange, TP: grey, and TP:BA: purple). PXRD patterns from the starting materials and TP:BA were given for comparison.

**Figure S4.1.5** Twice repeated time-resolved *in situ* (TRIS) PXRD measurements of the mechanochemical reaction with BA pre-milled (50 Hz, 5 min, 10 mm). The right traces indicate the phases present in each step (BA: orange, TP: grey, and TP:BA: purple). PXRD patterns from the starting materials and TP:BA were given for comparison.

**Figure S4.1.6** Twice repeated time-resolved *in situ* (TRIS) PXRD measurements of the mechanochemical reaction with TP pre-milled (50 Hz, 30 min, 8 mm). The right traces indicate the phases present in each step (BA: orange, TP: grey, and TP:BA: purple). PXRD patterns from the starting materials and TP:BA were given for comparison.

**Figure S4.1.7** Twice repeated time-resolved *in situ* (TRIS) PXRD measurements of the mechanochemical reaction with TP pre-milled (50 Hz, 5 min, 8 mm). The right traces indicate the phases present in each step (BA: orange, TP: grey, and TP:BA: purple). PXRD patterns from the starting materials and TP:BA were given for comparison.

**Figure S4.1.8** Twice repeated time-resolved *in situ* (TRIS) PXRD measurements of the mechanochemical reaction with TP pre-milled (50 Hz, 15 min, 8 mm). The right traces indicate the phases present in each step (BA: orange, TP: grey, and TP:BA: purple). PXRD patterns from the starting materials and TP:BA were given for comparison.

**Figure S4.1.9** Twice repeated time-resolved *in situ* (TRIS) PXRD measurements of the mechanochemical reaction with TP pre-milled (50 Hz, 1 min, 8 mm). The right traces indicate the phases present in each step (BA: orange, TP: grey, and TP:BA: purple). PXRD patterns from the starting materials and TP:BA were given for comparison.

**Table S4.1.1.** List of the induction and completion times for each set of parameters for the TP+BA system.

|  | **Pre-conditioning** | **Time /min** | **Time /min** | **Time /min** | **Mean value ± Std. Dev** |
| --- | --- | --- | --- | --- | --- |
| induction | no pre-milling | 25.0 | 28.0 | 19.0 | 24.0 ± 4.6 |
| completion |  | 50.0 | 50.0 | 42.0 | 47.3 ± 4.6 |
| induction | TP: 50Hz, 1min, 8mm ball | 20.3 | 22.0 | 0 | 21.15 ± 1.2 |
| completion |  | - | - | - | - |
| induction | TP: 50Hz, 5min, 8mm ball | 14.0 | 14.0 | 0 | 14.0 ± 0.0 |
| completion |  | 27.0 | 25.0 | 0 | 26.0 ± 1.4 |
| induction | TP: 50Hz, 15min, 8mm ball | 10.0 | 6.0 | 0 | 8.0 ± 2.8 |
| completion |  | 23.0 | 15.0 | 0 | 19 ± 5.7 |
| induction | TP: 50Hz, 30min, 8mm ball | 9.0 | 8.0 | 0 | 8.5 ± 0.7 |
| completion |  | 10.0 | 16.0 | 0 | 13.0 ± 4.2 |
| induction | TP: 50Hz, 5min, 10mm ball | 6.7 | 5.7 | 0 | 6.2 ± 0.7 |
| completion |  | 17.4 | 15.4 | 0 | 16.4 ± 1.4 |
| induction | TP: 50Hz, 30min, 10mm ball | 2.5 | 2.6 | 0 | 2.55 ± 0.1 |
| completion |  | 5.3 | 8.0 | 0 | 6.65 ± 1.9 |
| induction | BA: 50Hz, 5min, 10mm ball | 25.0 | 16.0 | 0 | 20.5 ± 6.4 |
| completion |  | 38.0 | 32.0 | 0 | 35.0 ± 4.2 |
| induction | BA: 50Hz, 30min, 10mm ball | 16.0 | 25.0 | 21.0 | 20.7 ± 4.5 |
| completion |  | 36.0 | 42.0 | 39.0 | 39.0 ± 3.0 |

Whilst prolonged pre-activation does lead to a continued decrease in the time-to-completion for the mechano-cocrystallisation of TP+BA, Figure S4.1.10 (orange), we were able to identify an ‘optimal’ pre-activation time to achieve the fastest overall reaction kinetics, Figure S4.1.10 (grey). To do so, we considered the total time needed to achieve completion of the TP+BA reaction (sum of pre-milling time and time-to-completion). We find that pre-activation for only 5 minutes leads to the overall shorted reaction time for the TP+BA cocrystallisation.

**Figure S4.1.10** Optimisation of mechanochemical kinetics for the mechano-cocrystallisation of TP+BA. The time of reaction (grey: sum of pre-milling time and time-to-completion; orange: only time-to-completion) are plotted against the pre-milling time, using data for pre-activation with an 8mm ball, from Table S4.1.1.

**S4.2 TRIS-PXRD of TP + NIC**

To obtain a semi-quantitative description of the transformation rates of the TP + NIC system under ball milling conditions, we performed a single peak integration of the powder diffraction data. This integration was done using Bragg reflections that were unique to the corresponding phase and isolated from other Bragg reflections. This corresponds to a Bragg reflection at $q\approx$ 10.5 nm^-1^ for TP, $q\approx$ 9.0 nm^-1^ for NIC, and $q\approx$ 9.6 nm^-1^ for the TP:NIC cocrystal.

Ball milling reaction rates were performed using non-activated and pre-activated reagents at 50 Hz using a 10 mm diameter milling ball. Corresponding datasets are provided in Figures S4.2.1-S4.2.3, showing duplicate experiments. A summary of the major rate events is given in Table S4.2.1.

**Figure S4.2.1** Twice repeated time-resolved *in situ* (TRIS) PXRD measurements of the mechanochemical reaction with no pre-milling. The right traces indicate the phases present in each step (NIC: pink, TP: grey, TP:NIC: green). PXRD patterns from the starting materials and TP:NIC were given for comparison.

**Figure S4.2.2** Twice repeated time-resolved *in situ* (TRIS) PXRD measurements of the mechanochemical reaction with TP pre-milled (50 Hz, 30 min, 10 mm). The right traces indicate the phases present in each step (NIC: pink, TP: grey, TP:NIC: green). PXRD patterns from the starting materials and TP:NIC were given for comparison.

**Figure S4.2.3** Twice repeated time-resolved *in situ* (TRIS) PXRD measurements of the mechanochemical reaction with NIC pre-milled (50 Hz, 30 min, 10 mm). The right traces indicate the phases present in each step (NIC: pink, TP: grey, TP:NIC: green). PXRD patterns from the starting materials and TP:NIC were given for comparison.

**Table S4.2.1.** List of the determined times for induction and completion of each set of parameters for the TP:NIC system.

|  | **Pre-conditioning** | **Time /min** | **Time /min** | **Mean value ± Std. Dev** |
| --- | --- | --- | --- | --- |
| induction | no pre-milling | 13.3 | 4.3 | 8.8 ± 6.4 |
| completion |  | 85.0 | 78.3 | 81.7 ± 4.7 |
| induction | TP: 50Hz, 30min, 10mm ball | 2.5 | 1.6 | 2.1 ± 0.6 |
| completion |  | 50.0 | 33.3 | 41.7 ± 11.8 |
| induction | NIC: 50Hz, 30min, 10mm ball | 3.6 | 4.0 | 3.8 ± 0.2 |
| completion |  | 50.0 | 51.8 | 50.9 ± 1.3 |

**S4.3 TRIS-PXRD of ANT + CAF**

To obtain a semi-quantitative description of the transformation rates of the ANT + CAF system under ball milling conditions, we performed a single peak integration of the powder diffraction data. This integration was done using Bragg reflections that were unique to the corresponding phase and isolated from other Bragg reflections. This corresponds to a Bragg reflection at $q\approx$17.1 nm^-1^ for ANT, $q\approx$8.4 nm^-1^ for CAF, and $q\approx$18.2 nm^-1^ for the CAF:ANT (I) cocrystal. The formation of CAF:ANT (II) was not included.

Ball milling reaction rates were performed using non-activated and pre-activated reagents at 50 Hz using a 10 mm diameter milling ball. Corresponding datasets are provided in Figures S4.3.1-S4.3.3, showing duplicate experiments. A summary of the major rate events is given in Table S4.3.1. Remarkably, the pre-activation of ANT leads to the formation of a different polymorphic form of ANT:CAF, Fig S4.3.3. The different polymorph of ANT:CAF requires prolonged milling (3 h, 50 Hz, 8 mm) to be obtained without pre-milling, S4.3.4.

**Figure S4.3.1** Twice repeated time-resolved *in situ* (TRIS) PXRD measurements of the mechanochemical reaction with no pre-milling. The right traces indicate the phases present in each step (CAF: pink, ANT: blue, CAF:ANT (I): green). PXRD patterns from the starting materials and CAF:ANT (I) were given for comparison.

**Figure S4.3.2** Twice repeated time-resolved *in situ* (TRIS) PXRD measurements of the mechanochemical reaction with CAF pre-milled (50 Hz, 30 min, 10 mm). The right traces indicate the phases present in each step (CAF: pink, ANT: blue, CAF:ANT (I): green). PXRD patterns from the starting materials and CAF:ANT (I) were given for comparison.

**Figure S4.3.3** Thrice repeated time-resolved *in situ* (TRIS) PXRD measurements of the mechanochemical reaction with ANT pre-milled (50 Hz, 30 min, 10 mm). The right traces indicate the phases present in each step (CAF: pink, ANT: blue, CAF:ANT (II): yellow). PXRD patterns from the starting materials and CAF:ANT (II) were given for comparison.

**Figure S4.3.4** PXRD measurement of mechanochemical cocrystalliaztion of CAF:ANT (50 Hz, 8 mm, 3 h) without pre-milling of reagents.

**Table S4.3.1.** List of the determined times for induction and completion of each set of parameters for the CAF:ANT system.

|  | **Pre-conditioning** | **Time /min** | **Time /min** | **Time /min** | **Mean value ± Std. Dev** |
| --- | --- | --- | --- | --- | --- |
| induction | no pre-milling | 18.3 | 20.0 | 0 | 19.2 ± 1.2 |
| completion |  | 65.0 | 81.6 | 0 | 73.3 ± 11.8 |
| induction | CAF 50Hz 30min 10mm | 10.0 | 15.0 | 0 | 12.5 ± 3.5 |
| completion |  | 75.0 | 70.0 | 0 | 72.5 ± 3.5 |
| induction | ANT 50Hz 30min 10mm | 9.3 | 7.3 | 9.3 | 8.7 ± 1.2 |
| completion |  | 26.7 | 36.7 | 33.0 | 32.1 ± 5.1 |

**S5 SCANNING ELECTRON MICROSCOPY ANALYSIS**

To understand the effect that pre-milling had on our starting reagents, we studied samples of non-milled and pre-milled (conditions) reagents by scanning electron microscopy (SEM). The SEM images for each of starting materials are given in Figure S5.1-S5.10.

**Figure S5.1** SEM images of theophylline in non-activated/ non-milled (left) and activated/milled (right) condition.

**Figure S5.2** SEM images of theophylline in non-activated/ non-milled (left) and activated/milled (right) condition.

**Figure S5.3** SEM images of benzamide in non-activated/ non-milled (left) and activated/milled (right) condition.

**Figure S5.4** SEM images of benzamide in non-activated/ non-milled (left) and activated/milled (right) condition.

**Figure S5.5** SEM images of nicotinamide in non-activated/ non-milled (left) and activated/milled (right) condition.

**Figure S5.6** SEM images of nicotinamide in non-activated/ non-milled (left) and activated/milled (right) condition.

**Figure S5.7** SEM images of caffeine in non-activated/ non-milled (left) and activated/milled (right) condition.

**Figure S5.8** SEM images of caffeine in non-activated/ non-milled (left) and activated/milled (right) condition.

**Figure S5.9** SEM images of anthranilic acid in non-activated/ non-milled (left) and activated/milled (right) condition.

**Figure S5.10** SEM images of anthranilic acid in non-activated/ non-milled (left) and activated/milled (right) condition.

**S6 BRUNAUER-EMMETT TELLER (BET) ANALYSIS**

It has been proposed that surface area has a significant influence on the reaction rate of mechanochemical transformations. To investigate pre-activation had its effect on reaction rates by an increase in surface area of the reactants, we measured the effect that pre-activation had on the surface area of starting materials by BET analysis, Table S6.1. We do not see any obvious correlation between the increase in surface area and the effect that pre-activation has on reaction rates.

**Table S6.1** The results of BET analysis on non-activated and pre-activated reagents. The weight of the measured sample is given, alongside the measured surface area. We report also the gas pressure range over which BET measurements were performed ($\frac{p}{p_{o}}$).

| **sample** | **weight / g** | **BET surface area / m²g^-1^** | **p/p_0_ range** |
| --- | --- | --- | --- |
| Theophylline | 1.3088 | 1.1 | 0.05-0.19 |
| Theophylline, pre-milled | 0.7545 | 2.1 | 0.05-0.22 |
| Nicotinamide | 1.1485 | 0.2 | 0.05-0.22 |
| Nicotinamide, pre-milled | 0.9398 | 4.2 | 0.03-0.16 |
| Benzamide | 1.3856 | 1.0 | 0.03-0.16 |
| Benzamide, pre-milled | 0.9499 | 4.0 | 0.16-0.29 |
| Caffeine | 3.6245 | 0.8 | 0.05-0.20 |
| Caffeine, pre-milled | 2.2920 | 5.5 | 0.05-0.20 |
| Anthranilic Acid | 3.1704 | 0.2 | 0.05-0.20 |
| Anthranilic Acid, pre-milled | 2.8696 | 3.0 | 0.05-0.20 |

**S7 REFERENCES**

[1]	M. Drakopoulos, T. Connolley, C. Reinhard, R. Atwood, O. Magdysyuk, N. Vo, M. Hart, L. Connor, B. Humphreys, G. Howell, S. Davies, T. Hill, G. Wilkin, U. Pedersen, A. Foster, N. De Maio, M. Basham, F. Yuan, K. Wanelik, “I12: the Joint Engineering, Environment and Processing (JEEP) beamline at Diamond Light Source” *J Synchrotron Rad* **2015**, *22*, 828–838.

[2]	I. Zizak, “mySpot: a versatile microfocussing station for scanning methods at BESSY II” *J. Large Scale Res. Facilities* **2016**, *2*, A101.

[3]	G. I. Lampronti, A. A. L. Michalchuk, P. P. Mazzeo, A. M. Belenguer, J. K. M. Sanders, A. Bacchi, F. Emmerling, “Changing the game of time resolved X-ray diffraction on the mechanochemistry playground by downsizing” *Nat Commun* **2021**, *12*, 6134.

[4]	J. Filik, A. W. Ashton, P. C. Y. Chang, P. A. Chater, S. J. Day, M. Drakopoulos, M. W. Gerring, M. L. Hart, O. V. Magdysyuk, S. Michalik, A. Smith, C. C. Tang, N. J. Terrill, M. T. Wharmby, H. Wilhelm, “Processing two-dimensional X-ray diffraction and small-angle scattering data in DAWN 2” *J Appl Cryst* **2017**, *50*, 959–966.

[5]	S. Brunauer, P. H. Emmett, E. Teller, “Adsorption of Gases in Multimolecular Layers” *J. Am. Chem. Soc.* **1938**, *60*, 309–319.

[6]	G. Fagerlund, “Determination of specific surface by the BET method” *Mat. Constr.* **1973**, *6*, 239–245.

[7]	E. ISO, “9277, Determination of the specific surface area of solids by gas adsorption using the BET method” *European Committee for Standardization (CEN), Brussels, Belgium* **2010**.

[8]	S. J. Clark, M. D. Segall, C. J. Pickard, P. J. Hasnip, M. I. J. Probert, K. Refson, M. C. Payne, “First principles methods using CASTEP” *Z. Kristallogr. Cryst. Mater.* **2005**, *220*, 567–570.

[9]	J. P. Perdew, K. Burke, M. Ernzerhof, “Generalized Gradient Approximation Made Simple” *Phys. Rev. Lett.* **1996**, *77*, 3865–3868.

[10]	S. Grimme, “Semiempirical GGA-type density functional constructed with a long-range dispersion correction” *J. Comput. Chem.* **2006**, *27*, 1787–1799.

[11]	S. Grimme, J. Antony, S. Ehrlich, H. Krieg, “A consistent and accurate *ab initio* parametrization of density functional dispersion correction (DFT-D) for the 94 elements H-Pu” *J. Chem. Phys.* **2010**, *132*, 154104.

[12]	S. Grimme, S. Ehrlich, L. Goerigk, “Effect of the damping function in dispersion corrected density functional theory” *J Comput Chem* **2011**, *32*, 1456–1465.

[13]	E. Caldeweyher, J.-M. Mewes, S. Ehlert, S. Grimme, “Extension and evaluation of the D4 London-dispersion model for periodic systems” *Phys. Chem. Chem. Phys.* **2020**, *22*, 8499–8512.

[14]	Y. Ebisuzaki, P. D. Boyle, J. A. Smith, “Methylxanthines. I. Anhydrous Theophylline” *Acta Crystallogr C Cryst Struct Commun* **1997**, *53*, 777–779.

[15]	Q. Gao, G. A. Jeffrey, J. R. Ruble, R. K. McMullan, “A single-crystal neutron diffraction refinement of benzamide at 15 and 123 K” *Acta Crystallogr B Struct Sci* **1991**, *47*, 742–745.

[16]	F. Fischer, M. U. Schmidt, S. Greiser, F. Emmerling, “The challenging case of the theophylline–benzamide cocrystal” *Acta Crystallogr C Struct Chem* **2016**, *72*, 217–224.

[17]	G. E. Hardy, W. C. Kaska, B. P. Chandra, J. I. Zink, “Triboluminescence-structure relationships in polymorphs of hexaphenylcarbodiphosphorane and anthranilic acid, molecular crystals, and salts” *J. Am. Chem. Soc.* **1981**, *103*, 1074–1079.

[18]	C. W. Lehmann, F. Stowasser, “The Crystal Structure of Anhydrous β‐Caffeine as Determined from X‐ray Powder‐Diffraction Data” *Chem. Eur. J.* **2007**, *13*, 2908–2911.

[19]	N. Madusanka, M. D. Eddleston, M. Arhangelskis, W. Jones, “Polymorphs, hydrates and solvates of a co-crystal of caffeine with anthranilic acid” *Acta Crystallogr B Struct Sci Cryst Eng Mater* **2014**, *70*, 72–80.

[20]	K. N. Jarzembska, A. A. Hoser, R. Kamiński, A. Ø. Madsen, K. Durka, K. Woźniak, “Combined Experimental and Computational Studies of Pyrazinamide and Nicotinamide in the Context of Crystal Engineering and Thermodynamics” *Cryst. Growth Des.* **2014**, *14*, 3453–3465.

[21]	F. Fischer, D. Lubjuhn, S. Greiser, K. Rademann, F. Emmerling **2016**, DOI 10.5517/CCDC.CSD.CC1KG7Y6.
